# The Influence of AlN Intermediate Layer on the Structural and Chemical Properties of SiC Thin Films Produced by High-Power Impulse Magnetron Sputtering

**DOI:** 10.3390/mi10030202

**Published:** 2019-03-22

**Authors:** Nierlly Galvão, Marciel Guerino, Tiago Campos, Korneli Grigorov, Mariana Fraga, Bruno Rodrigues, Rodrigo Pessoa, Julien Camus, Mohammed Djouadi, Homero Maciel

**Affiliations:** 1 Centro de Ciência e Tecnologia de Plasmas e Materiais—PlasMat, Instituto Tecnológico de Aeronáutica, São José dos Campos 12228-900, SP, Brazil; marcielguerino@yahoo.com.br (M.G.); moreiratiago22@gmail.com (T.C.); bruno.manzolli@gmail.com (B.R.); rspessoa@ita.br (R.P.); homero@ita.br (H.M.); 2 Space Research and Technology Institute, Acad. G. Bonchev Str. Bl.1, 1113 Sofia, Bulgaria; kgrigoro@abv.bg; 3 Instituto de Ciência e Tecnologia, Universidade Federal de São Paulo, São José dos Campos 12231-280, SP, Brazil; 4 Instituto Científico e Tecnológico, Universidade Brasil, Rua Carolina Fonseca 235, São Paulo 08230-030, Brazil; 5 Institut des Matériaux Jean Rouxel IMN, UMR 6502, Université de Nantes, 2 rue de La Houssinière, BP 32229, 44322 Nantes CEDEX, France; jcamus@gmail.com (J.C.); abdou.djouadi@cnrs-imn.fr (M.D.)

**Keywords:** high-power impulse magnetron sputtering (HiPIMS), silicon carbide, aluminum nitride, thin film, Rutherford backscattering spectrometry (RBS), grazing incidence X-ray diffraction (GIXRD), Raman spectroscopy

## Abstract

Many strategies have been developed for the synthesis of silicon carbide (SiC) thin films on silicon (Si) substrates by plasma-based deposition techniques, especially plasma enhanced chemical vapor deposition (PECVD) and magnetron sputtering, due to the importance of these materials for microelectronics and related fields. A drawback is the large lattice mismatch between SiC and Si. The insertion of an aluminum nitride (AlN) intermediate layer between them has been shown useful to overcome this problem. Herein, the high-power impulse magnetron sputtering (HiPIMS) technique was used to grow SiC thin films on AlN/Si substrates. Furthermore, SiC films were also grown on Si substrates. A comparison of the structural and chemical properties of SiC thin films grown on the two types of substrate allowed us to evaluate the influence of the AlN layer on such properties. The chemical composition and stoichiometry of the samples were investigated by Rutherford backscattering spectrometry (RBS) and Raman spectroscopy, while the crystallinity was characterized by grazing incidence X-ray diffraction (GIXRD). Our set of results evidenced the versatility of the HiPIMS technique to produce polycrystalline SiC thin films at near-room temperature by only varying the discharge power. In addition, this study opens up a feasible route for the deposition of crystalline SiC films with good structural quality using an AlN intermediate layer.

## 1. Introduction

Silicon carbide (SiC) has been proven to be a promising material for microelectronic applications due to its excellent physical and electronic properties, such as high surface hardness, wide bandgap, and high thermal conductivity at low and high temperatures [1,2,3,4,5,6]. These outstanding properties make it an attractive material for the development of harsh-environment devices such as Micro-Electro-Mechanical Systems (MEMS) and power electronics [1,2,7,8,9]. In a recent article, Dinh et al. showed an on-chip SiC MEMS device for efficient thermal management [10]. Furthermore, the strain effect in a highly doped 3C–SiC-on-glass substrate for mechanical sensors was also recently reported by Phan et al. [11]. The 3C–SiC bridges were also investigated under the consideration of Joule heating [12].

For microelectronic device applications, it is desirable for SiC thin films to be grown on Si substrates because their manufacturing processes are based on Si microfabrication technology, which is compatible with standard industrial processes [8,13,14,15]. It is difficult to grow high-quality crystalline SiC (c-SiC) films on Si substrates at low temperatures (<300 °C) due to a large mismatch between their lattice constant (about 20%) and thermal expansion coefficients (about 8%), which usually affects the final properties of the grown material [16]. In order to reduce these effects, an intermediate or buffer layer may be added. For this purpose, aluminum nitride (AlN) thin film is frequently used since it presents minimum mismatching in the lattice constant (less 1%) with SiC, and has a similar thermal expansion coefficient [17,18,19,20]. 

Meguro et al. investigated the formation of a SiC interfacial buffer layer on AlN/Si substrates at a low temperature by low-pressure chemical vapor deposition (LPCVD) [17]. Nakazawa et al. reported the epitaxial growth of SiC films on an AlN layer on Si (100) substrates by ultralow-pressure chemical vapor deposition. Jeong et al. investigated the Raman scattering characteristics of 3C–SiC films deposited on AlN/Si substrates using the atmosphere pressure chemical vapor deposition (APCVD) technique [19]. Huang et al. demonstrated the formation of SiC quantum dots (SiC QDs) on AlN films using low-frequency inductively coupled plasma (LF-ICP)-assisted magnetron sputtering [20]. To our knowledge, the study here presented is the first to report the growth of high-power impulse magnetron sputtering (HiPIMS) of SiC films on AlN/Si substrates.

The achievement of good crystallinity in the SiC thin films is a desirable feature since it influences different material properties [21]. As the SiC thin films deposited at low temperature grow in amorphous or nanocrystalline structures, post-treatment such as annealing, to improve the material crystallinity, is necessary. Although there are several well-known techniques for synthesizing SiC thin films, their composition and final properties may vary considerably with the applied method [3,19]. Low-pressure plasma-based techniques have been extensively investigated, particularly those that allow the deposition at near-room temperatures, such as plasma-enhanced chemical vapor deposition (PECVD) and magnetron sputtering [1,3,22,23,24,25]. Along with the magnetron sputtering derivations, the high-power impulse magnetron sputtering (HiPIMS) technique appears to be very attractive due to its ability to generate high-density plasmas and a high degree of ionization of the sputtered atoms [26,27,28,29,30,31]. These properties allow sufficient energy for the rearrangement of atoms/molecules during the growth of the film, thus facilitating the formation of crystalline phases. Some reports have demonstrated that, depending on the deposition parameters and target composition, around 50–90% of the sputtering atoms are in an ionized state [24,28,30]. This occurs because of the mechanism in which the HiPIMS power supply applies the power over the magnetron target for generating the plasma, namely high-power pulses, low frequency, and low duty cycles (lower than 10%) [28,30,31,32]. Interesting reviews on HiPIMS were written by Sarakinos et al. [30] and Gudmundsson et al. [31]. 

Although the HiPIMS source is applied in the synthesis of various metals and semiconductor materials, there is a clear lack of studies focusing on the growth of SiC thin films using this technique. The studies related to this topic are focused on Ti–Si–C and SiCN films using HiPIMS [26,33]. In the work of Alami et al. [33], the effect of processing parameters such as gas pressure, substrate geometry, and distance of the target substrate on some properties of the as-deposited Ti–Si–C film was investigated. They observed that the Ti–Si–C film quality could be improved by the HiPIMS technique [33]. Pusch et al. performed a comparison between SiCN films deposited with different target configurations and techniques, i.e., radiofrequency (RF), direct current (DC), and HiPIMS [26]. Leal et al. deposited SiC thin films on Si substrates by HiPIMS using a SiC target [34]; however, only amorphous films were obtained. In this article, we explore the structural and chemical properties of polycrystalline SiC films grown at room temperature on Si and AlN/Si substrates by the HiPIMS technique. The composition, chemical bonding, structure, and crystallinity of the samples were investigated by Rutherford backscattering spectrometry (RBS), Raman spectroscopy, and grazing incidence X-ray diffraction (GIXRD). 

## 2. Materials and Methods

### 2.1. Deposition Method

SiC thin films were grown onto polished p-type Si (100) wafers, either with or without an AlN layer, via HiPIMS in a high-vacuum chamber with a background pressure of 6 × 10^−6^ Torr. More details about the HiPIMS reactor can be found elsewhere [34,35]. The working pressure of the argon gas (99.999%, White-Martins, São José dos Campos, Brazil) was maintained at 3 mTorr for a corresponding flow rate of 20 sccm (standard centimeter cubic per minute). The target was a commercial high-purity SiC (99.5%, Kurt J. Lesker Company, Jefferson Hills, PA, USA) with a diameter of 4 inches. For film growth, the applied power from the HiPIMS power supply (HIP^3^ 5 kW, Solvix SA, Villaz-Saint-Pierre, Switzerland) was 200 W and 400 W. In all cases, the duty cycle was fixed at 5%, frequency at 500 Hz, and pulse time at 100 μs. To remove the target surface contaminants, a pre-sputtering time of 200 W for 10 min was applied. In addition, the substrate holder was maintained at a floating potential, whereas the deposition time and the target-substrate distance were fixed at 60 min and 60 mm, respectively.

The AlN/Si substrates were provided by the “Institut des Matériaux Jean Rouxel in Nantes University”. In these substrates, the sputtered AlN intermediate layer had a thickness of 1300 nm and a (002) crystallographic orientation. For more details, see References [36,37]. 

### 2.2. Characterization Techniques

Rutherford backscattering spectrometry (RBS) was used to measure the elemental composition, stoichiometry, and the thickness of the as-deposited SiC thin films. The measurements were performed with a pelletron accelerator using 2 MeV 4He^+^ beam with a particle detector positioned at 170° from the incident beam. The RBS spectra were analyzed using the computer code RUMP (RBS analysis package) developed by L. R. Doolittle from Cornell University [38]. To verify the accuracy of the RBS thickness measurements and the thickness uniformity, mechanical profilometry (P-7 Stylus Profiler, KLA Tencor, Milpitas, CA, USA) measurements were performed.

The crystallinity of the SiC films was inferred from GIXRD with incidence angles (ω) of 1.0°, 1.5°, and 2.0° using an X-ray diffractometer (PW1830/1840, Philips, Amsterdam, The Netherlands) with CuK_α_ radiation. For Raman spectroscopy measurements, a model 2000 Renishaw system (Renishaw, Wotton-under-Edge, UK), equipped with an Ar ion laser (514.5 nm) was used. Raman spectra were obtained at room temperature in the range of 400–1800 cm^−1^.

## 3. Results and Discussion

### 3.1. Chemical Composition and Stoichiometry

**Figure 1:**
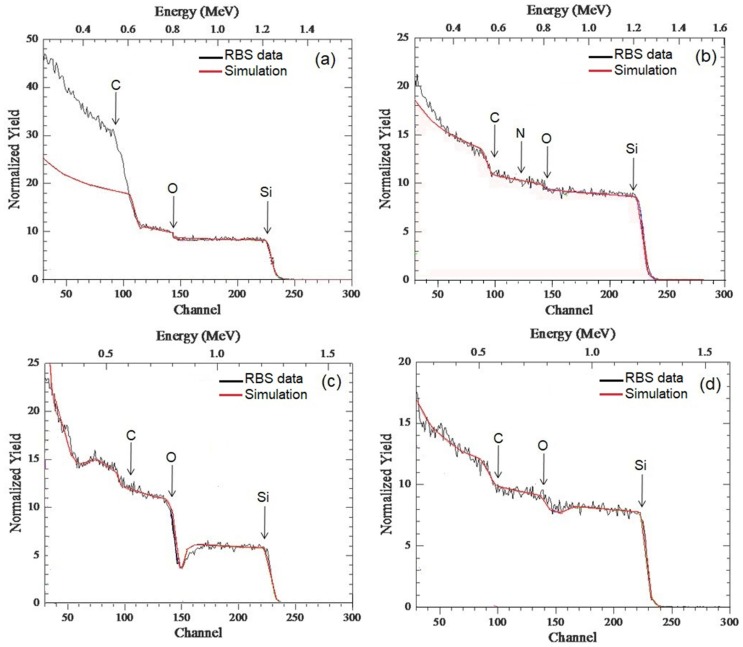
Experimental and simulated Rutherford backscattering spectrometry (RBS) spectra of the SiC films deposited on (**a**) Si substrate at 200 W; (**b**) Si substrate at 400 W; (**c**) AlN/Si substrate at 200 W and; (**d**) AlN/Si substrate at 400 W.

Figure 1 shows the experimental and simulated RBS spectra of the as-deposited SiC thin films on Si and AlN/Si substrates for the 200 W and 400 W conditions. Table 1 summarizes the results of the RBS spectra analysis. 

Figure 1a depicts the spectrum of SiC deposited on Si at 200 W and the simulation reveals a film with a total thickness of approximately 1200 nm, with a highly non-homogenous elemental distribution throughout the film depth. To better visualize the variation of the stoichiometry throughout the film depth, the simulation comprised five sublayers which are identified in Table 1. The top layer’s stoichiometry comprised 260 nm of pure SiC with less than 13% oxygen. The middle part (approximately 400 nm) consisted of SiC with 10% excess carbon. The next layer with a thickness of 250 nm had about 50% carbon excess and a substantial drop in the oxygen content was observed. The subsequent layer of 170 nm was fully stoichiometric, followed by the last layer of 145 nm adjacent to the Si surface, where 10% carbon excess was found. When investigating the incorporated oxygen in the first layers of the SiC film, Medeiros et al. observed the unintentional doping of SiC_x_N_y_ thin films by oxygen contamination coming from the vacuum environment of the magnetron co-sputtering system [35]. In this work, RBS results showed that all samples contained significant amounts of oxygen (up to 16%). Further, X-ray photoelectron spectroscopy (XPS) results showed that most of this oxygen is located in the film surface [35]. These results corroborate with the RBS analysis presented in Figure 1a. In addition, Pomaska et al. presented studies on the unintentional doping by oxygen contamination where they demonstrated that the oxygen incorporation was influenced the microstructural, electronic, and optical properties of the SiC films [39]. It has been shown that oxygen incorporation during film deposition increases the crystallinity of SiC films, consistent with findings observed in this work.

For the SiC grown on the Si substrate at 400 W (Figure 1b), the analysis of the RBS spectra indicated that the total film thickness was around 1500 nm. The film exhibited a pure and stoichiometric composition of SiC throughout the entire depth, although two zones could be distinguished as presented in Table 1. Beyond the SiC, SiO_2_, and SiN phases, there were O and N contaminants. This sputtering condition resulted in a heterogeneous film composition with variable elemental depth distributions. In general, the higher power deposition energy, as in this case, leads to Ar ions striking onto the film surface with high energy, which contributes to the formation of chemical phases. Of course, if different impurities act as film constituents, they are involved in the film composition forming stable bonds (SiO_2_; SiN). 

From the thickness results of the SiC films grown at 200 W (1200 nm) and 400 W (1500 nm), it is possible to observe that although the applied power is twice as high, there was a small increase in the deposition rate for SiC films on the Si substrate. In conventional sputtering processes, the deposition rate of the SiC film increases linearly with the sputtering power [23,24]. In general, HiPIMS exhibits different growth mechanisms and lower deposition rates than those observed for conventional sputtering processes [30,31,40]. Different effects have been considered to explain the differences between DC and HiPIMS deposition rates. There are three main reasons considered [41]: (i) the less-than-linear increase of the sputtering yield with increasing ion energy, ion return to the target, and self-sputtering; (ii) ion return to the target and self-sputtering; and (iii) changes due to greater film density, limited sticking, and self-sputtering on the substrate.

For the SiC film deposited on AlN/Si at 200 W, the total film thickness was around 930 nm (Figure 1c). The film composition was rather homogenous and consisted of 56% pure SiC, while the remaining 44% of the film was composed of C and O in the bulk of the film. The intermediate layer of AlN consisted of 1300 nm thick sub-stoichiometric AlN with 5% less nitrogen, resulting in some point defects. Note that in this case the substrate change provided the growth of a high stoichiometric SiC film. Relative to film thickness, it is evidenced from data presented in Table 1 that the change of Si with AlN/Si substrate promoted the decrease in the thickness of the SiC film. Although sputtering processes have deposition rates that are independent of the substrate type, the film nucleation process and consequent crystallization and compaction are dependent. Nivedita et al. confirmed some of these observations when depositing RF-sputtered Fe–Ga thin films on MgO, quartz, and Si substrates [42]. Indeed, the next topic shows that the crystallization of SiC is improved for films deposited on Si. Crystalline films tend to have greater roughness and even porosity in comparison with amorphous films, which consequently increases the final thickness [43].

The SiC thin film deposited on AlN/Si at 400 W (Figure 1d) exhibited a high percentage of purely stoichiometric SiC film, with the presence of C and O in volume. However, for this condition the estimate of the thickness by RBS was limited due to the loss of the energy via scattering albeit within certain limits, e.g., above channel n° 90 (Figure 1d), the thickness could be estimated as being around 1360 nm. Ultimately, the elemental depth distribution throughout the film thickness was uniform, which made the present method and processing conditions very useful for the achievement of high-quality SiC thin film deposition.

Finally, from the results in Table 2, it was possible to observe that the calculated deposition rates of the SiC films were in agreement with the profilometry measurements. With regard to the deposition rates measured by profilometry, and where the film thicknesses were measured at different points during the formation of the film, it was possible to evaluate the uniformity of the film thickness, which exhibited a 3% variation throughout the substrate. In fact, the greater the target–substrate distance in processes performed by magnetron sputtering, the better the uniformity of the film formed, where a distance of 60 mm was used. With regard to the film morphology, in previous work [44] Atomic force microscopy (AFM) analyses of the SiC/AlN/Si film and the AlN/Si film were performed, showing films with rough surfaces and with grain sizes smaller than 100 nm.

### 3.2. Structural Analysis

**Figure 2:**
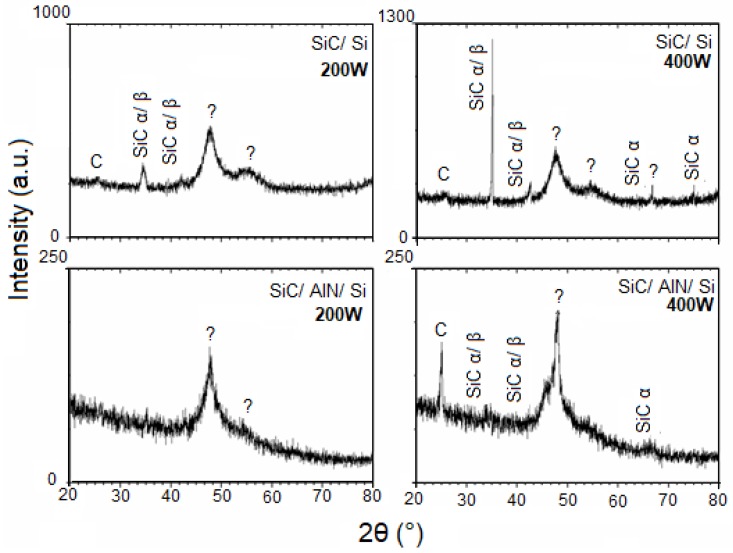
Grazing incidence X-ray diffraction (GIXRD) patterns of the SiC thin films at a grazing angle of 1.0°.

**Figure 3:**
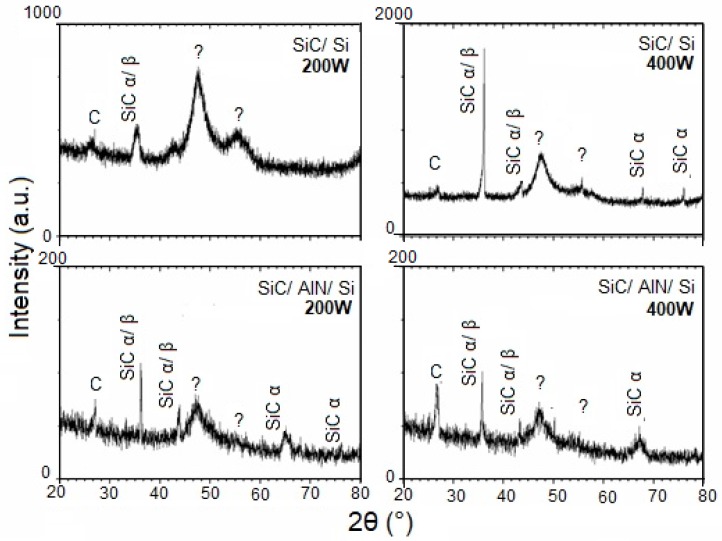
GIXRD patterns of the SiC thin films at a grazing angle of 1.5°.

**Figure 4:**
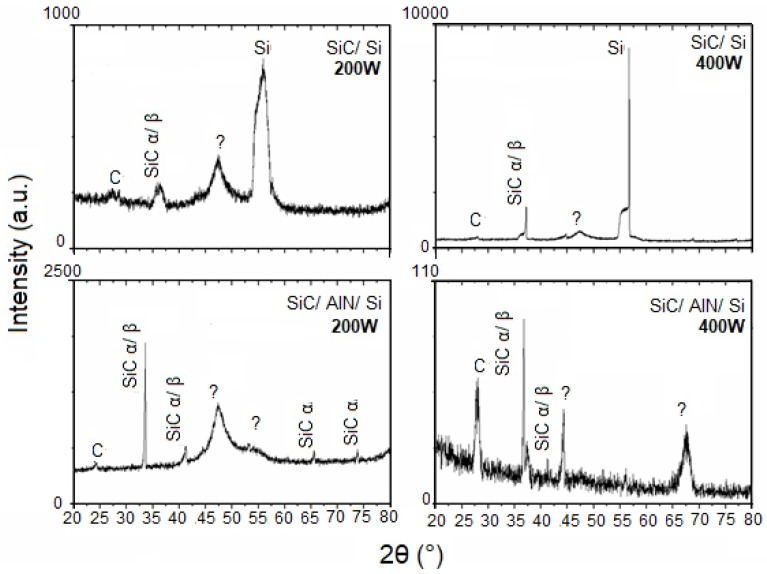
GIXRD patterns of the SiC thin films at a grazing angle of 2°.

Figure 2, Figure 3 and Figure 4 show the patterns of grazing incidence angles of 1.0°, 1.5°, and 2.0°, respectively. The Bragg reflections suggest the existence of α and β SiC nanocrystalline structures. Although the patterns exhibit the SiC phase, it is not possible to determine which of the SiC phases are present because some diffraction peaks of α and β SiC might overlap [45]. The carbon phase at ~25° was also visible and confirmed the RBS results indicating an excess of C (Table 1). Lastly, two broad peaks at ~47° and 55° were assigned as unidentified. While some studies have attributed these peaks to the SiC polymorph phase [46,47], others often define them as being C or Si phases [48,49,50]. When comparing the results from the GIXRD with an incidence angle of 2° (Figure 4) with the smaller angle results, the variation of the crystalline phases with the depth of the film was clearly noted.

In addition, Figure 2 suggests the existence of SiC nanocrystalline structures achieved without substrate heating. By varying the GIXRD incidence angle, the resulting film could be analyzed in depth. A comparison of the patterns between SiC/Si and SiC/AlN/Si for both angles of incidence (1° and 1.5°), revealed that the phase observed at approximately 36°, using the 1.5° GIXRD as the incidence angle, no longer existed in the pattern obtained with the smallest angle (1.0°). This result pointed to the existence of phase and crystallinity variations with depth. 

The film deposited on the AlN layer showed dislocation of the SiC peak between 35 and 36° in the GIXRD patterns. This dislocation may be attributed to the following reasons: (i) film stress; (ii) interference of the substrate (SiC/Si and SiC/AlN interface); or (iii) residual stress. More studies are necessary to better understand this observation. 

### 3.3. Raman Spectroscopy

**Figure 5:**
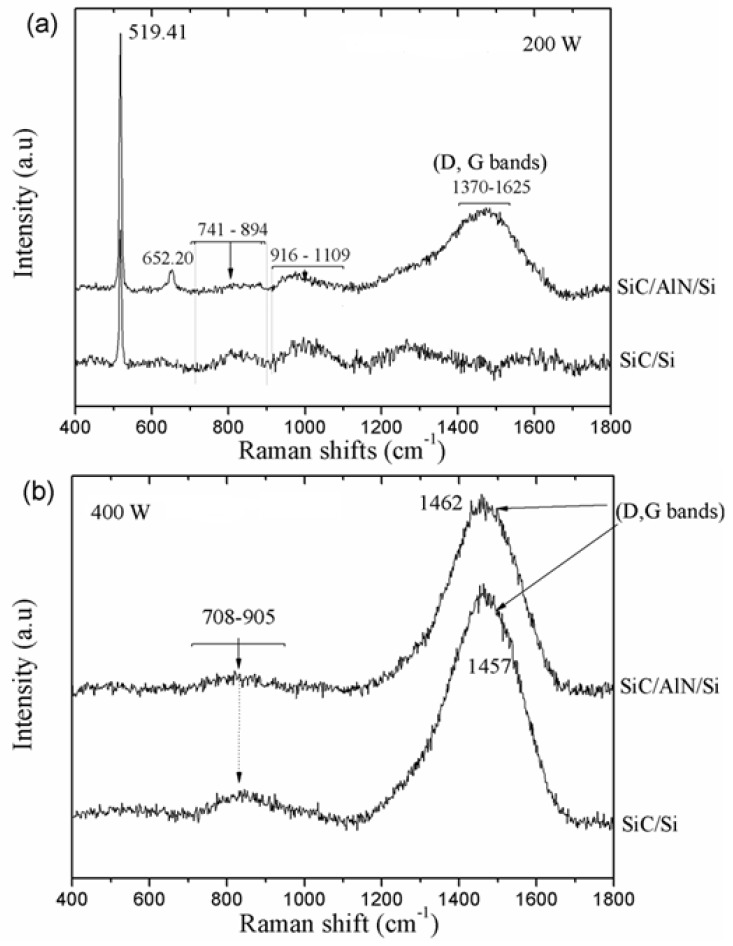
Raman spectra of SiC thin films on both substrates: (**a**) as-deposited at 200 W and (**b**) as-deposited at 400 W.

Figure 5 shows the results of Raman spectroscopy used to identify the bonds present in the films. The Raman spectra for the SiC films deposited at 200 W in both substrates (with and without the AlN layer) are presented in Figure 5a, showing a very visible and well-defined Si peak at 519.41 cm^−1^. Since the difference in thickness between both films was small, the substrate had an accentuated influence. In addition to Si, the SiC film deposited on the AlN layer showed (i) a peak relative to AlN at ~652.20 cm^−1^; (ii) peaks for SiC and Si in the regions between 741–894 cm^−1^ and 906–1109 cm^−1^, respectively; (iii) and a broad carbon band at 1370–1625 cm^−1^. Except for the AlN peak, the SiC/Si spectrum exhibited signals at similar regions to that of the SiC/AlN/Si spectrum. However, the regions relative to SiC and Si were more visible, and the C band region had a more explicit separation in two peaks (D and G bands), but with a low definition of the disorder band. The D band was attributed to the disorder or polycrystalline carbon and the G band to the graphite-like carbon [19,50]. 

For 400 W (Figure 5b), both substrates exhibited similar behavior following the deposition of SiC films. In these spectra, the substrate signal was not detected and only peaks corresponding to carbon at 1457 and 1462 cm^−1^ were evident. With the exception of the spectrum of the SiC film at 200 W of SiC/Si, where the peak of the C band was not overlapped, all spectra of the “as-deposited” SiC film showed overlapping C, D, and G bands between ~1457 and 1462 cm^−1^. Ferrari and Robertson [51] reported that for C–C bands, the Raman spectrum was influenced by factors such as disorder, clustering of the sp^2^ phase, the presence of sp^2^ C-rings or C-chains, and the ratio of sp^2^ to sp^3^ (I(D)/I(G)). Thus, with an increasing disorder of the C phase, the G peak position can be moved, and the D and G peaks will therefore overlap [19,50,51].

## 4. Conclusions

The influence of an AlN intermediate layer on the structural and chemical properties of HiPIMS SiC films grown on Si substrates was investigated using RBS, Raman spectroscopy, and GIXRD. The effect of the applied power (200 W and 400 W) was also considered. It was observed that the HiPIMS of SiC films can exhibit a complex growth mechanism and, depending on the process parameters, leads to the formation of films with an inhomogeneous composition throughout the depth of the Si substrate and a homogeneous composition for the AlN/Si substrate. This was verified by GIXRD using three different incidence angles (1.0°, 1.5°, and 2.0°) which, besides confirming the RBS results, also evidenced the variation of crystallinity with the depth of the film. Raman spectroscopy analysis indicated the presence of Si–C bonds and that the C–C bond region was separated into two peaks (D and G bands), but with a low definition of the disorder band. In summary, the results demonstrated that the HiPIMS technique and the use of an AlN intermediate layer allowed for the deposition of crystalline SiC films of good quality, without the need for substrate heating, with approximately 1.5 μm (at 400 W) in only 60 min, i.e., at a deposition rate of 25 nm/min. The good chemical and physical properties of the HiPIMS SiC films deposited on AlN/Si substrates highlights their potential benefits in nanotechnological applications. Indeed, we recently proposed the thermal decomposition of SiC thin films using a CO_2_ laser beam without a vacuum chamber for graphene synthesis. The use of an AlN layer proved to be important because it reduces the thermal stress between SiC and Si materials [44]. Other applications will be the subject of further work.

## Figures and Tables

**Table 1:** Results of the RBS analysis.

Sample	No. of Layers	Composition by Layer ^1^	Layer Thickness
SiC/Si 200 W	5	SiC stoichiometry with less than 13% oxygen. SiC with about 10% excess carbon. SiC with about 50% excess carbon. SiC stoichiometry. SiC with about 10% excess carbon.	1. 260 nm 2. 400 nm 3. 250 nm 4. 170 nm 5. 145 nm
SiC/Si 400 W	2	SiC—86%; SiO_2_ phase—4% dispersed in that volume; O—5%; N—5%. SiC—50% and SiN—50%.	1. ~900 nm 2. ~600 nm
SiC/AlN/Si 200 W	2	SiC stoichiometry—56%; C solid state and O contamination in 44%. AlN layer stoichiometry.	1. ~930 nm 2. ~1300 nm
SiC/AlN/Si 400 W	2	SiC stoichiometry—80% with 20% C solid state and O contamination in volume. AlN layer stoichiometry.	1. ~1360 nm 2. ~1300 nm

^1^ Layer 1 refers to the layer at the top of the film.

**Table 2:** Deposition rate of the SiC films.

Sample	Power (W)	Deposition Rate—RBS (nm/min)	Deposition Rate—Profilometer (nm/min)
SiC/Si	200	20.0	14.0 ± 0.3
SiC/Si	400	25.0	19.6 ± 0.4
SiC/AlN/Si	200	15.5	12.5 ± 0.4
SiC/AlN/Si	400	22.7	24.0 ± 0.5
